# Investigation of microstructural symmetry in regional zones of human multi-rooted teeth using optical, electrical, and ion diffusion methods

**DOI:** 10.14440/jbm.2024.0122

**Published:** 2025-08-15

**Authors:** Vladimir Mikhailovich Zolotarev

**Affiliations:** Department of Physical Optics and Spectroscopy, Faculty of Photonics, ITMO University, Saint-Petersburg 197101, Russia

**Keywords:** Symmetry, Optical anisotropy, Dentinal tubules, Growth zones

## Abstract

**Background:**

Dentin is a mineralized tissue characterized by a complex network of dentinal tubules, whose arrangement significantly influences the mechanical and physiological properties of teeth.

**Objective:**

This study investigated the influence of the microstructural symmetry of dentinal tubules in two orthogonal sections of the crown of human molar and premolar teeth.

**Methods:**

The effect of symmetry on the microstructure of dentin sections was studied for two orthogonal sections of the human molar and premolar crown. The symmetry of local zones of tooth sections was first examined using a set of methods: optical, electrical, and ion-diffusion techniques. The methods used have different resolutions and display both the general properties of the dentin structure and the properties that are specifically revealed by an individual method. It is shown that dentinal tubules originate from the center of the cusps of both molars and premolars, forming S-shaped fiber bundles presenting an axial-radial symmetry.

**Results:**

The dentinal tubules were shown to originate from the center of the cusps in both molar and premolar teeth, forming S-shaped fiber bundles with axial-radial symmetry. These bundles were arranged along axes, extending from the pulp toward the centers of the cusps of the tooth crown. Within these zones, distinct optical patterns resembling conoscopic figures in the form of a “Maltese cross” were observed. This indicates a highly ordered architecture composed of optically anisotropic uniaxial tubules. The optical data were correlated well with findings obtained by electrometric and ion diffusion methods, including dentinal tubule staining.

**Conclusion:**

The polarization optical is a valuable tool for studying various regional organizations of dentinal tubules in dentin.

## 1. Introduction

The study of human teeth is intriguing from multiple perspectives. The unique morphological characteristics of the tooth crown can facilitate individual identification and, through chemical composition analysis, help determine geographic origin.^1^,^2^ This is particularly important in archeology, where teeth often represent the only surviving remains, enabling researchers to study evolutionary transformations in a species. Research into the morphological details of crown shape in multi-rooted teeth has revealed heuristic patterns that offer insights into tooth development and evolution,^3^-^6^ particularly the relationship between crown and root properties.^5^ Minor variations in crown morphology and the number of roots are among the most evolutionary responsive dental features. The tooth crown is extensively used to establish developmental principles linking genotype to phenotype.^7^ Despite the abundance of literature, the historical formation of the mammalian dental system remains relatively understudied. This is reflected in the ongoing lack of consensus regarding the evolutionary development of multi-rooted teeth during phylogenesis. The transformation of simple conical reptilian teeth into complex, multi-rooted mammalian teeth with complex crown relief remains a subject of scientific debate.^5^,^8^,^9^ Given that the structure of dentin is intimately tied to crown shape, studying the symmetry elements involved in dentinogenesis may provide additional insights into the developmental properties and evolutionary features of the tooth.^4^

The concept of symmetry and the role of tooth evolution have been discussed in the literature at the morphological level, particularly in relation to the structure of the outer part of the molar crown.^5^ Symmetry in living organisms is associated with their adaptation to the surrounding environment.^10^-^14^ There are different points of view on the development and formation of tooth types during the evolutionary transition from a simple conical shape.^5^-^8^ The properties of dentinal tubules, which form the basis of tooth structure, have been studied in detail using various physical methods;^14^-^18^ however, the mutual organization of dentinal tubules, which determines the shape of a particular tooth, has not yet been examined from the angle of symmetry. Assuming that the relief of the tooth crown evolved from simple conical teeth, the dentin structure near the tubercles of multi-rooted teeth may contain fragments of symmetry and asymmetry. Given the optically anisotropic properties of dentin, the study of such structures, preserving elements of symmetry and asymmetry, is well amenable to polarized light techniques used in the optics of anisotropic media. The high sensitivity of the polarization method allows for the analysis of symmetry–asymmetry in images, facilitating the study of genotypic and phenotypic features of tooth development. This renders the optical polarization method an important addition to traditional techniques, such as electrical conductivity and ionic diffusion. The ability to visualize the organizational structure of tubules near the crown section may provide further insights into the causes underlying the formation of multi-rooted teeth.

This work aimed to study the visualization of the distribution of tubules in regional zones of the molar crown by examining two principal axial sections of human teeth using optical polarization, alongside traditional methods of electrical conductivity and ion diffusion.

## 2. Dentin and research methods

### 2.1. Properties and structure of dentin

The structure of dentin is complex and hierarchical, consisting of micro- and nanostructures of peritubular dentin (PTD), from which the dentinal tubules are directly formed, and intertubular dentin (ITD), which fills the space between the tubules. PTD is a relatively dense, mineralized tissue surrounding the tubules of the coronal dentin of the tooth. It is believed that the border between PTD and ITD is structurally homogeneous.^19^ Dentinal tubules follow an S-shaped path from the pulp to the dentinoenamel junction. The number of tubules per unit area is about 54,000/mm^2^ in the inner dentin, 30,000/mm^2^ in the middle dentin, and 8,000/mm^2^ in the outer dentin. The diameter of the tubules measures about 1.9 μm in the inner dentin, 1.4 μm in the middle dentin, and 1.2 μm in the outer dentin.^20^ The lumen of many tubules contains cellular extensions of odontoblasts, which are responsible for dentin growth. These cells occupy most of the space inside the tubules and regulate the movement of transudate, a fluid present in the dental pulp, through a system of distributed micro-openings. Dentinal tubules also contain nerve fibers that respond to external stimuli. The odontoblastic process is accompanied by a bundle of nerve fibers located in the concavities on its surface, extending 50–160 μm.^21^,^22^ A-beta and A-delta nerve fibers are responsible for dentin sensitivity; their endings are situated in the pulp-dentin interface. Several hypotheses have been proposed to explain the transmission of stimuli through dentin. These include direct nerve stimulation, dentin receptors, ion diffusion, and the hydrodynamic mechanism. The most commonly accepted mechanism of nerve activation being associated with dentin sensitivity is the hydrodynamic mechanism.^23^-^25^ The presence of abundant pores and cracks in PTD indicates that PTD does not primarily dictate the mechanical strength of dentin, but rather plays an important role in transporting pulp fluid via the tubules to support dentin’s viability as a living tissue.^23^ The biological consistency of dentin across mammalian species has enabled the extension of experimental models to animals in both *in vitro* and *in vivo* studies, allowing comparisons with human tooth properties.^24^-^28^ Several studies noted that, in terms of various indicators (*e*.*g*., shape, crown size), human molars are comparable to their swine counterparts, which have a large-sized thick enamel, and a similar crown morphology. However, pig molars develop much faster than human teeth. Due to regional differences in molar tooth properties, many studies focused specifically on certain tubercle areas using a large number of experimental specimens.^20^,^26^-^28^ Therefore, the teeth of domestic pigs serve as a convenient model biosystem for addressing practical questions in odontology and studying the biomineralization process of the human tooth.^29^-^32^

### 2.2. Optical properties of dentinal tubules

The characteristics of dentin, a biological material from which the tooth is made, are associated with its anisotropy, which arises from the inherent properties of the dentinal tubules. The anisotropy of these tubules and their structural organization leads to the anisotropy observed in several tooth properties, including permeability and sensitivity (which are related to ionic conductivity,^16^,^33^ as well as optical,^34^-^41^ mechanical,^42^,^43^ and electrical properties.^44^-^48^ Dentin tubules can be likened to uniaxial crystals with properties similar to hydroxyapatite (HAp). HAp crystals belong to the hexagonal syngony type and are optically uniaxial and negative. The optical axis of HAp coincides with the crystallographic c-axis, which is aligned with the crystal’s longest dimension. Nanocrystals of HAp that form part of the structure of human PTD have an average length of 36.00 ± 1.87 nm, a width of 25.57 ± 1.37 nm, and a thickness of 9.76 ± 0.69 nm. Each nanocrystal is a flattened hexagonal prism with an average width-to-thickness ratio of 2.61. The long axis of the HAp nanocrystal is in alignment with the axis of the dentinal tubule. The orderly packing of individual HAp nanocrystals within the tubules determines the optical properties of the tubules, which mimic those of a set of uniaxial nanocrystals whose optical axis runs along the tubule. The average refractive index of dentin is *n* = 1.50 ± 0.02,^49^ with a birefringence of approximately Δn = n_e_ – n_o_ = 0.005. Natural crystals exhibit slight dichroism, giving them a yellowish hue. As the relative refractive index of the tubules n_ITD_/n_PTD_ = (1.45–1.5/1.59) is <1, light illuminated at a grazing incidence angle will propagate along the tubules under conditions of total internal reflection.

To reduce background noise due to scattering when studying the optical properties of anisotropic disperse systems such as tubules, the use of polarized light is advantageous. The interaction of polarized light with ordered structural zones in the section produces cone-shaped figures, enabling experimental visualization of symmetry features in regional zones of the tooth section.

### 2.3. Sample preparation

The study involved 36 intact teeth—molars and premolars of the upper and lower jaws—extracted from patients aged 18 to 40 years for orthodontic reasons. Indications for extraction included common orthodontic issues, such as crowding, protrusion, and malocclusion. All tooth extractions were performed anonymously at the educational department of the Faculty of Dentistry of the N.I. Pirogov Medical University (St. Petersburg, Russia) for clinical orthodontic indications. The extracted teeth were anonymized and classified as biological waste; therefore, in accordance with the ethical principles of the institution, no additional patient consent was required for their use in the study. The average crown size of the second molars was 9.4 ± 0.24 mm for the upper jaw and 10.2 ± 0.24 mm for the lower jaw. The average size of the third molar crown was virtually identical to that of the second molars. All tooth samples used in the study were free of carious or non-carious defects. Samples were prepared in two main orientations: horizontal sections (perpendicular to the tooth axis) and vertical sections (parallel to the tooth axis).

For disinfection and preservation, the teeth were stored in a 40% formalin solution. Horizontal sections were prepared as plane-parallel plates approximately 1,000 μm thick.^36^,^37^ Some molar samples were also sectioned vertically through the cuspal tips, typically along the diagonal axis of the tooth.

### 2.4. Investigation of dentin using the polarization method

In areas with an ordered arrangement of anisotropic dentinal tubules, local regions of the tooth exhibit axial symmetry. For sections cut perpendicular to the tooth axis, a central convergence point of dentinal tubule bundles can be visualized using crossed polarizers. Light passing through such axially symmetric bundles is periodically extinguished by the analyzer, producing a system of concentric light and dark rings intersected by a dark cross. The ring diameter depends on the section’s thickness and the tubule arrangement within the bundle, which reflects symmetry or asymmetry in the visualized images.^37^ For the analysis, thin sections were placed in a humidified chamber situated between the polarizer and the analyzer. The optimal orientation of the section was established visually, ensuring that the polarization vector of the incident light was orthogonal to the axis of the tubule bundle in the cusp zone. Reproducibility of visual observations was ensured by fixing the section’s position relative to the polarization vector (E-vector) of the output of the polarizer.

### 2.5. Investigation of dentin permeability by ion diffusion

To determine section permeability, we used dye ions that diffused inside the dentinal tubules under external hydraulic pressure.^33^,^49^,^50^ Tubule permeability reflects the ability to transport pulp fluid, which is involved in tooth biogenesis. Dentin permeability primarily depends on the thickness of dentin within a section (*i*.*e*., the length of the tubules) and the tubule diameter. Given that tubules are shorter, more numerous, and have a larger diameter closer to the pulp, deep dentin is less effective as a pulp barrier compared to superficial dentin. Regional differences in dentin permeability are evident at the periphery, with values being higher near the dentin-enamel layer and lower in the center of the crown section. The hydraulic conductivity of dentin in the radial direction from the center of the section decreases with increasing distance from the pulp and with increasing dentin thickness. Dentin permeability can vary by 3–10 times within just a few millimeters across different regional zones of the tooth.^50^-^52^ Patterned images (depicting template, regularity, and model) resulting from specialized staining of sections have been observed for a long time. However, a unified classification of such zones has yet to be established.^53^ Therefore, to further study the regional (local) properties of the tooth, it is important to identify the features of the structural organization of the tubules. In this context, the use of complementary analytical methods remains relevant.

### 2.6. Evaluation of regional optical patterns

The reproducibility of visual patterns across different section samples depends on individual variations in tooth structure, particularly the microstructure of dentinal tubules in regional crown areas. In the comparison of 36 molar samples, it was noted that for a small percentage (~7%) of samples, within regional zones approximately 1–1.5 mm, the characteristic image patterns (crosses and concentric rings) were nearly absent. This indicates a spatially disordered or non-linear (tortuous) course of dentinal tubules in those zones. In contrast, typical optical patterns were found in approximately 75% of the samples within these regional zones: on average, four crosses (three complete) were seen in second molars, and five crosses (four complete) in third molars. The effectiveness of the crossed-polarizer method is influenced by significant attenuation of light as it passes through the ground section, particularly when the sample structure is homogeneous. In such cases, the contrast of the observed image is noticeably reduced. There are also limitations when studying older teeth, as ground sections from these samples typically exhibit low transparency and high light scattering, which further reduces the contrast of the observed pattern. The data presented in Table 1 demonstrate the visual results and reproducibility of three different methods applied to the study of regional zones in three different molars. A strong correlation is evident among the measurements of growth zones obtained using these three independent methods across the examined samples.

**Table 1:** Morphometric characteristics of the regional sections of the molar crown

Tubercle numbers	Growth zone area/tooth cross-section (relative units)		Size of growth zone/tooth cross-section (relative units)			Diameter of the inner (open) ring/diameter of the growth zone (relative units)
		
	Optic	Ion diffusion	Optic	Ion diffusion	Electrometric	Ion diffusion
1	3.0	4.7	0.24	0.20	0.18	0.45
2	1.3	4.4	0.18	0.14	0.16	0.45
3	2.0	3.7	0.16	0.09	0.14	0.45
4	1.1	2.0	0.10	0.09	0.14	0.45
5	-	≤1	-	0.04	-	0.45

Sources:^33^,^40^,^47^.

## 3. Results and discussion

### 3.1. Section of the grind perpendicular to the tooth axis

**Figure 1:**
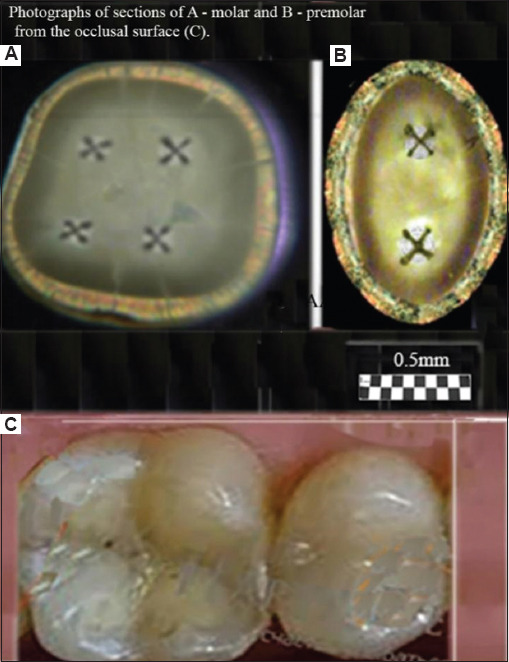
Photographs (patterns) of sections obtained by transmission in crossed polarizers for a molar (A) and a premolar (B), cut across the tooth axis (horizontal section). The scheme used to obtain these patterns is shown in Figure 3. (C) Photographs of the molar and premolar from the occlusal surface. A visual examination of the crowns of two different teeth clearly shows that the number of crosses corresponds to the patterns obtained using the optical polarization method. The illumination method for the tooth section is shown in Figure 5. Scale bars = 0.5 mm.

Images (patterns) of the structure of the horizontal section of the crown of the molar and premolar, obtained using crossed polarizers, are shown in Figure 1. Elements of the figure demonstrate the features of symmetry of the figures in the form of crosses and fragments of concentric rings, displaying characteristics of the structure in the regional areas of the section.

#### 3.1.1. Basic figures in the area of dental tubercles

**Figure 2:**
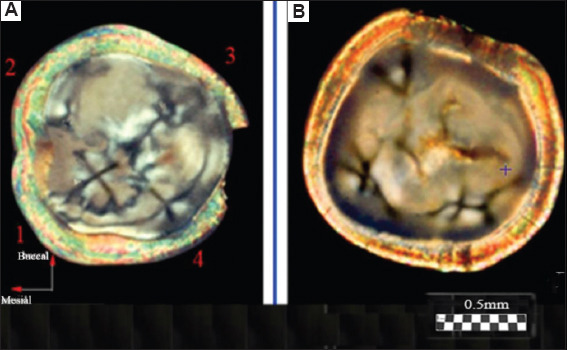
Photographs (patterns) of sections obtained in transmission using crossed polarizers for two different molars (A and B). The method of illuminating the tooth section is shown in Figure 5. A visual examination of the patterns reveals both common cross-shaped figures and clear differences. These differences illustrate the asymmetry in the structure of the tubular bundle within the peripheral growth zone. Scale bars = 0.5 mm.

As shown in Figure 1, four crosses are visible for the molar (a) and two crosses for the premolar (b); the number of crosses coincides with that of tubercles for the corresponding teeth (c). The most typical patterns for two molars are shown in Figure 2. The sizes of the figures resembling crosses correlate, in this regional area, with the height of the tooth tubercles. The peculiarities of the observed figures (symmetry–asymmetry) in sections of the crowns of three different molars (Figures 1 and 2) are associated with a disruption in the systemic orientation of the tubules in the regional growth zones that form the cusps of the tooth. Some tubules have an S-shaped configuration and differ in curvature in various parts of the crown. The curvature of the tubules in the center of the crown is usually depicted as stochastically curved branches diverging from the center. In contrast to this arrangement, which is characteristic of the center of the tooth, in the area of the molar cusps, the tubules are arranged systemically within a relatively narrow conical bundle. The density of tubules in dentin at the cusp location also differs significantly from the density of tubules beneath the occlusal fissure.^50^ This arrangement of tubules and their increased concentration allows for the observation of lightened zones in the cusp areas under crossed polarizers. It is in these zones that figures with elements of axial symmetry appear (Figure 1).

In the photograph of the tooth section (Figure 2), light growth zones are visible in the area of the molar cusps, often framed by relatively narrow dark lines. The light zones are caused by a set of isolated dentinal tubules organized in the form of a cone, with an axis directed from the pulp of the tooth and passing through the apex of the cusp. The brightness of the zones with cross-like figures differs noticeably from the overall yellow–green background and is usually most intense in the cusp area (Figures 1 and 2). The light beams propagating along the tubules have a small aperture in these bright, small zones, and the axes of these zones form an angle of approximately 25° with the normal to the surface of the section. The brightness of an individual zone increases significantly when observed along the axis of the beam. In some cases, in the center of the section, one can observe a low-contrast figure in the form of a “Maltese cross,” larger than similar figures seen in the bright regional zones adjacent to the molar tubercles. The initial visual observations under crossed polarizers^36^ allowed us to detect, for the 1^st^ time, fragments of a low-contrast large (~4–6 mm) “Maltese cross” in the center of the section, appearing within 2–3 quadrants and accompanied by concentric light and dark rings. This indicates uniaxial symmetry of the tubules in the center of the molar section. Such samples with fragments of a large “Maltese cross” in the center of the section were occasionally encountered in our studies. Nevertheless, all these data indicate the presence of elements of symmetry in the formation of the tubule bundle at the center of the section.

#### 3.1.2. Additional figures in the form of “dark lines”

In the process of tooth formation, the so-called extended “dark lines” and darkened zones are of significant importance (Figure 2). These “dark lines” and darkened zones are mainly located between the dentin-enamel layer and the main growth zone in the center of the tubercle. Extended “dark lines” are formed by closely spaced bundles of small diameter (~100–350 μm), consisting of dentinal tubules with a cone angle of φ_1_ ≤ 1°. These narrow bundles are formed by a system of practically parallel anisotropic tubules, through which the S_0_ beam is dampened by the analyzer. At small image sizes, a figure in the form of a “Maltese cross” together with a system of rings cannot be formed due to the limited size of the optical beam. The image features (patterns) of the molar crown section structure, obtained by the polarization method, allow for comparison with similar structures obtained by other methods.

#### 3.1.3. Patterns of the molar section: Ion diffusion method

**Figure 3:**
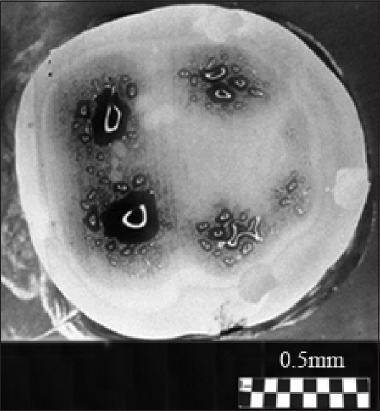
Photograph (pattern) of a molar section obtained using the ion diffusion method.^33^ A liquid containing dark blue dye was introduced into the pulp chamber under pressure. Dark areas of dentin in the region of the tubercles (above the pulp horns) are significantly more permeable than the superficial (unstained) dentin in the center of the section. White open rings appear in the center of the dark area, indicating regions where the dentin is impermeable to the dye. Scale bar = 0.5 mm.

**Figure 4:**
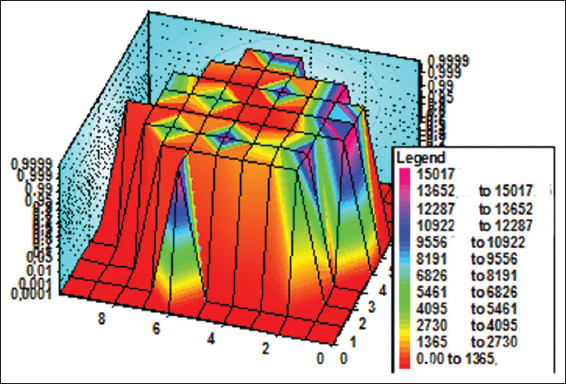
Patterns of resistance of a molar section with cross-section.^48^ The resistance scale is given in kΩ, with corresponding colors indicated on the left. For example, the presence of red color on the surface of the section indicates a defect. The graph is aligned along the Z-axis using a logarithmic scale. The numbers of points corresponding to the line-by-line scanning of the tooth section surface are indicated along the X and Y axes. The pattern clearly shows the symmetry of the two growth zones of the section.

As an example, Figures 1 and 2 compare with similar patterns obtained by independent methods: ion diffusion^33^,^50^-^52^ (Figure 3) and electrometric analysis^44^-^48^ (Figure 4). From the comparison of Figures 1-3, it follows that the narrow bundles of tubules observed as “dark lines” and darkened zones represent small local growth zones located around the main growth zones that form the tooth cusps.

The functional purpose of these small regional growth zones is to correct the relief during the formation of an individual tooth cusp. Analysis of the different thicknesses of the “dark lines” in the vicinity of the cusps, based on optical measurements, allows us to independently estimate the average size of the small growth zones, which is ≥100 μm. This is consistent with the data obtained with the diffusion method.

#### 3.1.4. Patterns of the molar section: Electrical resistance method

As shown in Figure 4, it is evident that the electrical resistance of dentin is lower in the tubercle area than in the central part of the tooth. For a single tubercle, the resistance value decreases as it moves from the periphery to the center. This trend in resistance within the tubercle indirectly suggests a higher axial symmetry at the center relative to the periphery. In some areas on the outer edge of the section, resistance is also lower, which indicates the presence of cracks in the enamel. The comparison of Figures 1-4, obtained using three different methods, revealed a correlation of the features of the pattern elements, particularly in the tubercle area of the tooth. The regional zones in the observed patterns are determined by the properties of the dentinal tubules in the crown portion of the molar tooth. The tubules are grouped in specific regions of the section relative to the center of the bundle. Moreover, the central part of these tubules exhibits a higher degree of organization, which is mirrored in the symmetry of the observed patterns. The permeability of dentin to liquid and its sensitivity to external factors depend on the diameter and organization of the tubules in the bundle. The most significant structural differences in tubule organization are observed in the crown area of the molar, where the tooth tubercles are located (Figure 3). Based on the darkened regions of the section, dentin appears more permeable to the stained liquid in these areas than in the light central part of the tooth.

#### 3.1.5. Optical model of the tooth section

For a more detailed study of its properties, when using the polarization method, it is advisable to construct an optical model of the tooth section. Based on such a geometric model, it is possible to calculate the path of rays inside the tooth section and compare the obtained data with experimental results.

When anisotropic tubules form a cone-shaped beam, the phase relationships of orthogonal rays (ordinary: So, extraordinary: Se) passing through such a cone at different distances from the center of the beam will exhibit a phase difference δ.^37^-^39^ As the rays propagate along the cone, the phase difference δ between the So and Se rays follows the rule of radial symmetry. It is essential to note that rays emerging from individual linear tubules at varying distances from the φ-axis and with azimuth α = 0–360° will be elliptically polarized.

**Figure 5:**
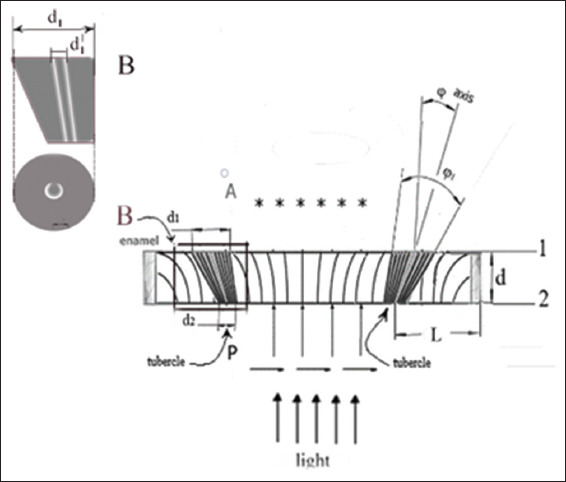
Schematic representation of the sectional view of a molar tooth showing the horizontal section in the area of the crown cusps. Notes: B: The structure of the bundle of dentinal tubules is shown to scale on the left—the dark area corresponds to typical dentinal tubules (which contain pulp fluid), and the transparent open ring corresponds to dentinal tubules presumably filled with nerve endings. d is the thickness of the section, L is the distance of the bundle from the border with the outer enamel of the tooth, φ is the angle of inclination of the axis of the bundle of tubules, φ^1^ is the angle of bundle alignment, d1 is the diameter of the main bundle of the growth zone in the area of the cusp on the upper plane of the section, d_2_ is the diameter of the main bundle in the area of the cusp on the lower plane, d_1_^1^ is the diameter of the open bundle of tubules in the center of the growth zone, which are filled with nerve endings. The thickness “t” of the internal open ring directly depends on the zone diameter d_1_. Depending on the value of d_1_, the thickness of the ring can range approximately from 8 to 80 μm. The ring break has a fixed orientation in the plane of the section. Additional parameters include: n is the refractive index of dentin (main), n^1^ is the refractive index of dentin with nerve endings (n ≈ n^1^), Δn is the birefringence, and N (φ) is the number of beam reflections in the tube. Horizontal arrows and asterisks indicate the positions of the polarization vectors during illumination, where P is the polarizer and A is the analyzer. The arrows at the bottom indicate the direction of the incident light beam on the section.

The magnitude of the phase shift δ for linear tubes will depend on the parameters: d, φ, α, n, Δn, and N(φ).^28^,^39^ If the tubules have non-linear dimensions, such as S-shaped forms or twists along the axis,^18^ then the phase difference δ will also be influenced by additional parameters: β (twist angle) and ρ (cone radius, ρ_max_ = d^1/2^). For a bundle of tubules with linear dimensions, the following quantities were used in the calculation of δ: d (section thickness), ρ (cone radius in the upper plane of the section), l (φ) (tubule length), β = 0, n = n_ITD_/n_PTD_ (relative refractive index of dentin), φ (ρ) (tubule inclination angle in the bundle), φ^1^ (cone angle), Δn ≈ 0.005 (birefringence of tubules, and N (φ), *i*.*e*., the number of beam reflections inside the tubules) (Figure 5). For these calculations, software was developed that allows one to demonstrate the influence of various parameters of anisotropic tubules organized in the form of an axisymmetric cone, the axis of which is located at a small angle to the incident light beam.^39^

Typical conditions used in the calculations included: β = 0, and the E-vector of the polarizer being perpendicular to the cone axis. In this case, only the So beam will be observed at the input of the anisotropic tubules, which will be quenched by the analyzer at the output of the cone. In the center of the calculated pattern, a cross is observed, the intersection of which coincides with the cone axis.

**Figure 6:**
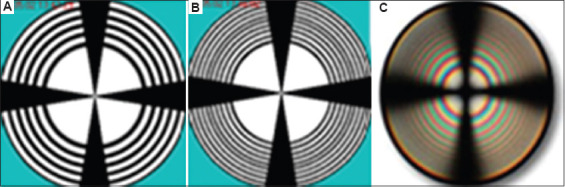
Interference pattern modeled for a molar section cut across the tooth axis (horizontal section), as shown in Figure 5. Monochromatic light (λ = 0.55 μm) is used, and the polarizer and analyzer are crossed. Inner diameter of the tubes: (A) −d_B_ = 0.1 μm, (B) −d_B_ = 1.5 μm, outer diameter of the tubules d_H_ = 3.5 μm, section thickness d = 1,000 μm, φ-10°, φ_1_ = 10°. Light rings represent the interference areas of the So and Se rays as they exit the tubules. Cross-shaped figures and dark zones indicate areas of attenuation of the So and Se rays. (C) Experiment: Classical conoscopic pattern in a converging beam of white light for a uniaxial crystal (d=1 cm) cut perpendicular to the optical axis.

**Figure 7:**
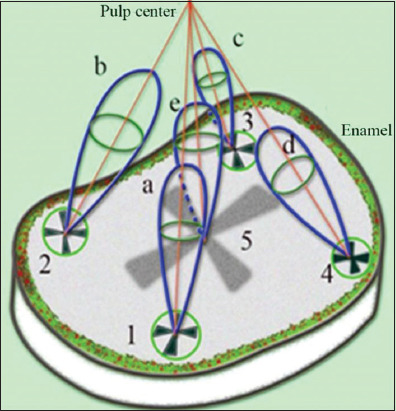
Diagram of a model of a molar section with an axisymmetric arrangement of tubules relative to the bundle axis in the area of the tubercles (1–4) and the center of the section (5). Brightness indicatrices (a, b, c, d, e) are shown for each zone corresponding to the tubercles (1–4) and the central cross (5), respectively. The brightness indicatrix “e” in the center of the section is significantly weaker. The illumination scheme for polarized light is shown in the opposite direction compared to that in Figure 5.

The shape of the cross in Figure 6 differs somewhat from the typical “Maltese cross” observed in experiments involving thick uniaxial single crystals. This is due to the fact that the light-quenching conditions for a system of anisotropic tubules distributed in an isotropic medium are observed only in the immediate vicinity of the main directions of the E-vectors of the polarizer and analyzer. Another characteristic feature of the calculated pattern appears in the form of dark and light rings, which the central cross intersects. The appearance of these rings is associated with the phase difference δ between the propagating So and Se beams. The phase difference δ (ρ,α) depends on the radius ρ and the azimuth α. The first light ring from the center corresponds to a phase difference of δ = 2π, resulting from the phase difference δ between the So and Se beams after passing through the tubules, and the additional π shift due to the orientation of the crossed polarizers. The first dark ring from the center corresponds to a phase difference δ = 2π + π, and so on. It should be noted that all the observed features of the described pattern are characteristic of an axisymmetric arrangement of a bundle of tubules in the shape of a cone. If the inclination angles of the tubules in the bundle are stochastic, only the central cross will be visible, and the rings will not appear. The presence of rings indicates an ordered arrangement of linear tubules around the cone axis (Figure 1A and B). The most contrasting cross-shaped figures are observed when the polarizers are crossed and the direction of the incident light’s E-vector is perpendicular to the axis of the truncated cone. Based on these calculations, Figure 7 shows a model of a molar tooth section with an axisymmetric arrangement of tubules^40^ in the area of the tubercles and the central part of the tooth.

The diagram for the tooth section model (Figure 7) shows that the axes corresponding to the bundles of tubules from four different growth zones of the second molar converge in the area of the tooth pulp.^27^ The varying inclination of the tubules in the central part of the tooth and near the growth zones of the molar tubercles leads to optical effects (Figures 1 and 2), which result from the disruption of symmetry in the regional zones.

#### 3.1.6. Visualization of figures in regional zones of thin section (experiment)

**Figure 8:**
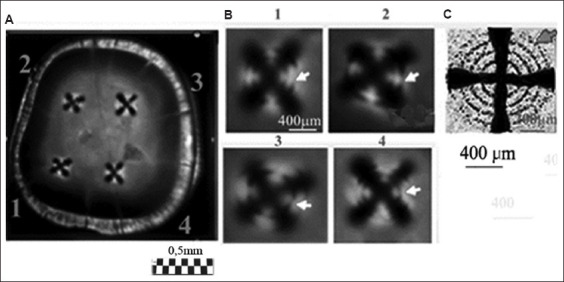
Presence of Maltese crosses in the vicinity of the tooth tubercles and rings within the local zones. (A) Photograph (pattern) of a section of the second molar, cut across the tooth axis and obtained in transmission using crossed polarizers, showing growth zones 1–4. Scale bar = 0.5 mm. (B) Enlarged images of growth zones 1–4. Arrows indicate interference rings surrounding the central parts of the crosses. The breaks in the interference rings indicate that the axisymmetric packing of the tubule bundles in zones 1–4 is mainly preserved near the center (*i*.*e*., the axis of the tubule bundle) and varies between the zones (1–4). Scale bar = 400 μm. (C) For comparison, a local zone of a premolar tooth^38^ is shown, exhibiting regular axisymmetric packing of linear tubules. Scale bar = 200 μm.

The appearance of a cross with rings in the center of the section is associated with the axisymmetric arrangement of the tubules in the central part of the section. An irregular arrangement of the tubules alters the systematicity of the function δ (ρ, α), and the magnitude of these disturbances depends on the distance ρ as it increases from the cone axis. From the example shown in Figure 8A and B, it is conspicuous that the magnitude of the phase deviation δ, for all values of ρ, is greatest for azimuths α corresponding to the positions of the diagonals within each of the four quadrants. It can be noted that the periodicity of the rings may not be preserved across adjacent quadrants (Figure 8B [1-4]). This may be associated with a disturbance in the angle of inclination of the tubules near a given quadrant or with local rotation of the S-shaped tubules in that quadrant. Concentric rings in the studied batch of sections were observed in only one, or two or three quadrants, in individual section samples, indicating a systematic arrangement of dentinal tubules in the vicinity of those specific quadrants. This, in turn, implies asymmetry in areas outside those quadrants.

A comparison of the sizes of regional zones of the molar section, based on data from different methods, is presented in Table 1. Table 1 shows a good correlation in the sizes of regional zones, although some differences are more pronounced in the smaller zones. These differences are likely due to methodological features and additional phenogenetic factors influencing the formation of tubercles in smaller zones of the tooth.

#### 3.1.7. Structure of a bundle of tubules and symmetry of figures (experiment)

**Figure 9:**
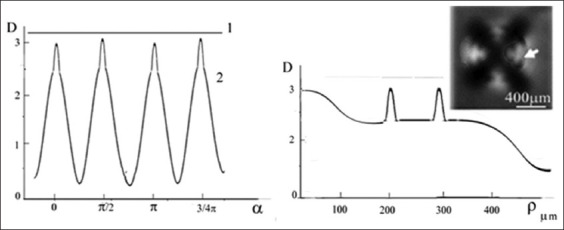
The periodic dependence of the azimuthal optical density D(α) and the radial optical density D(ρ). (A) Azimuthal dependence of optical density D(α) for ring No. 2. Note: 1 indicates theoretical calculation (Figure 6) and 2 represents experimental data (Figure 8^1^). The increase in D(α) near α = 0, π/2, π, ¾ π is due to imperfections in the tubule arrangement relative to the tubercle center. The position α = 0 corresponds to the orientation of the E-vector of the polarizer P. (B) Radial dependence of optical density D(ρ) for α = 0. The peaks on the D(ρ) curve correspond to the positions of the dark interference rings shown in the image above. Scale bar = 400 μm.

The features of the optical density *D* of the rings in the patterns, as show in Figure 8, allow us to plot graphs of the azimuthal dependence *D* (α) and radial dependence *D* (ρ) (Figure 9).

**Figure 10:**
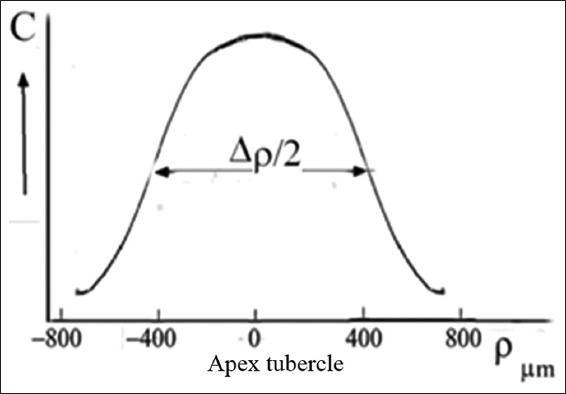
Distribution pattern of the concentration C(ρ) of tubules with a systemic orientation relative to the tubercle apices, obtained from the analysis of D(α) (Figure 8). At the edges of the half-width Δρ/2, the C(ρ) function shows stochastic variation with increasing distance from the tubule center.

Analysis of these graphs reveals the characteristics of the tubule distribution relative to the center of the tubercle. It seems that, in the vicinity of the tubercle center, the tubules are linear and exhibit higher symmetry relative to the apex of the tubercle (Figure 10).

#### 3.1.8. Features of the organization of tubules and symmetry of figures (experiment)

It is of note that there were samples in which, in the area of regional zones, figures other than crosses were observed (looking like shapes such as the letters V or X, unclosed eights, or a three-pointed star).^36^-^40^ The deformation of the cross shape may be ascribed to the specific arrangement of curved tubules. For example, the observed pattern may be associated with the presence of S-shaped tubules twisted along the axis. As can be inferred from Figure 2B, the twist angle increases smoothly with distance from the center of the figure. The presence of nearby local growth zones with similar symmetry may lead to the fusion of these zones, manifested as articulated centers forming crosses (Figure 2B). Different sizes of local growth zones in the vicinity of molar tubercles, obtained using the ion diffusion method, are shown in Figure 9.^16^,^33^

**Figure 11:**
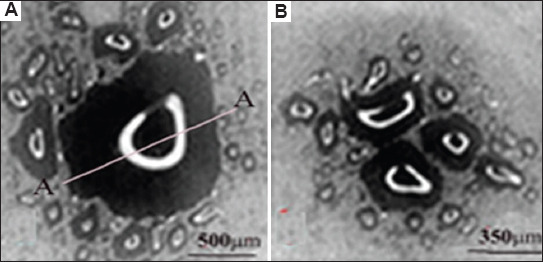
Growth zones (A and B) of a horizontal section of a molar (patterns) (Figure 3). Notably, the rupture areas of the unstained open rings are oriented in the same direction and correspond to dentin growth zones that are impermeable to the liquid dye. An important observation is that the white open rings in the centers of the growth zones share a consistent orientation, supporting the hypothesis that the tubules forming such structures play a role in adapting to external factors (*e*.*g*., environment, diet). Scale bars: A = 500 μm; B = 350 μm.

**Figure 12:**
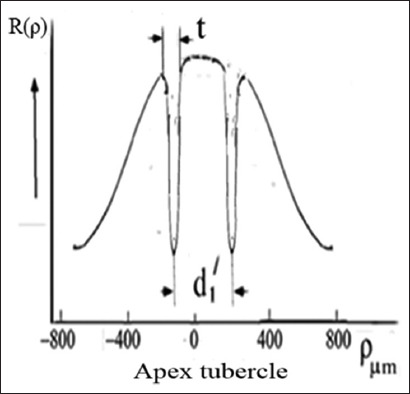
Schematic diagram of the resistance dependence R(ρ) in the cross-section near an individual tubercle, based on the correlation analysis of R(ρ) and the optical density D(ρ) in the tubercle region (section AA in Figure 11A**)**. The parameters d_1_^1^ and t represent the diameter and thickness, respectively, of the open transparent ring, which corresponds to high ionic conductivity in this zone of the tubercle.

Comparison of optical data with those obtained from the diffusion method reveals additional details in the form of open light rings, which are absent in the optical patterns (compare Figures 2 and 11). The presence of open light rings in the center of the growth zones (Figure 11) indicates the absence of dye ion diffusion in the tubules of that zone. Differences in ion diffusion ability between the center and periphery of the tubercle, along with differences in tubule symmetry, can lead to differences in electrical properties as well (Figure 12).

The course of the *R*(ρ) dependence is dictated by the features of the optical density function *D*(ρ) within the tubercle region (Figure 11A). Typical diameters of small growth zones range from approximately 100 to 350 μm, which, based on estimated calculations, corresponds to roughly 400–5,000 tubules within a small growth zone. The tubules in the region of open transparent rings with diameter *d*_1_^1^ (Figure 12) are uniformly oriented. Moreover, the position of the average center of the set of transparent broken rings within a single tubercle (Figure 11B) coincides with the center of the growth zone in the optical patterns, corresponding to the crosshair position in a specific zone (Figures 1 and 2). The dark area within the region of the open transparent rings (Figure 11) cannot be observed using the polarization method, as the optical properties of the tubules (n|) in this region do not differ significantly from those of the peripheral tubules (n) of the growth zone. However, these differences can be detected using electrical methods with high resolution (Figure 4). The consistency of these properties across the compared zones (Figures 1-3) suggests that the area covered by the open transparent ring may contain dentinal tubules sealed with nerve endings. The relative average diameter of the open ring zones remains constant across all growth zones. The thickness “t” of the open transparent rings varies only slightly across all tubercles, ranging from approximately 8 to 80 μm, which allows, by estimate, 2–16 tubules to be located in the cross-section of the transparent ring. The reasons for the breaks in the open transparent rings and their consistent orientation across different regional zones of one tooth are not yet fully understood. However, it may be assumed that the presence of such specialized tubules in the center of regional growth zones is logically related to nerve cells that regulate the growth of dentinal tubules and collectively form the relief shape of the tooth crown. At the same time, it is important to note that, according to biological theory, the observed symmetry of figures in the form of crosses within the regional zones of the tooth indicates a genetic type of dentinogenesis, whereas asymmetry in such figures is associated with a phenogenetic type of development. A clear example of phenogenetic development is demonstrated in experiments studying the surface modification of a domestic pig’s molar when fed soft versus hard feed. When fed coarse feed, a large number of sharp, small tubercles developed on the enamel crown surface. These were clearly recorded in the form of Maltese crosses in patterns observed using the polarization method.^38^,^39^ The average size of these growth zones was approximately 0.2–0.4 mm, and the crosses had a well-defined shape, indicating high symmetry in such small local growth zones. This modified crown structure allowed the pig to more effectively grind and crush coarse food.

### 3.2. Section of the grind parallel to the axis of the tooth

The optical scheme for observing a molar section in a selected orientation under transmission mode can be compared to methods used for studying fibers in an immersion medium. In this case, anisotropic fibers (dentinal tubules–PTD) are embedded in an immersion medium, namely ITD, which fills the spaces between the tubules. Given that the refractive indices of PTD and ITD are close to each other, such a composite system transmits light well and scatters it relatively weakly.

#### 3.2.1. Comparison of sections of different thicknesses

**Figure 13:**
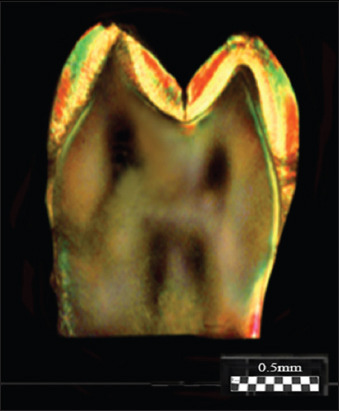
Photograph (patterns) of a molar section (thickness ≈ 1 mm) cut along the tooth axis (vertical section), obtained using crossed polarizers. Elongated dark fragments are visible, extending from the pulp toward the central part of the tooth tubercles. The illumination method used for the section is shown in Figure 5. Scale bar: 0.5 mm.

The use of crossed polarizers for a thinner section cut parallel to the tooth axis allows for the observation of a contrast pattern composed of light and dark fragments (Figure 13).

**Figure 14:**
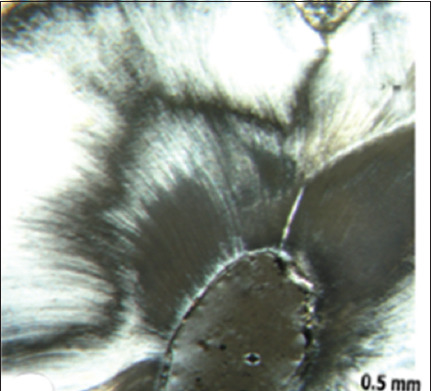
Fragment of a photo (pattern) of a molar section (thickness ≈ 0.03 mm) cut along the tooth axis (vertical section), obtained using crossed polarizers.^41^ The dark oval area in the lower part of the image corresponds to the pulp region. The small thickness of the layer in this area allows for complete light extinction under crossed polarizers. Scale bar: 0.5 mm.

For comparison, Figure 14 shows a photograph of a thinner molar section (d ≈ 0.03 mm). Comparing Figures 13 and 14 reveals that a thinner section produces a high-contrast pattern, which is due to a decrease in scattered light. In the thin section, structural details such as narrow black stripes and lightened areas are clearly visible; in the photo of Figure 13, these present as uniformly colored dark and light gray regions.

#### 3.2.2. Bending of tubules and connection with observation of contrast in dentin areas

**Figure 15:**
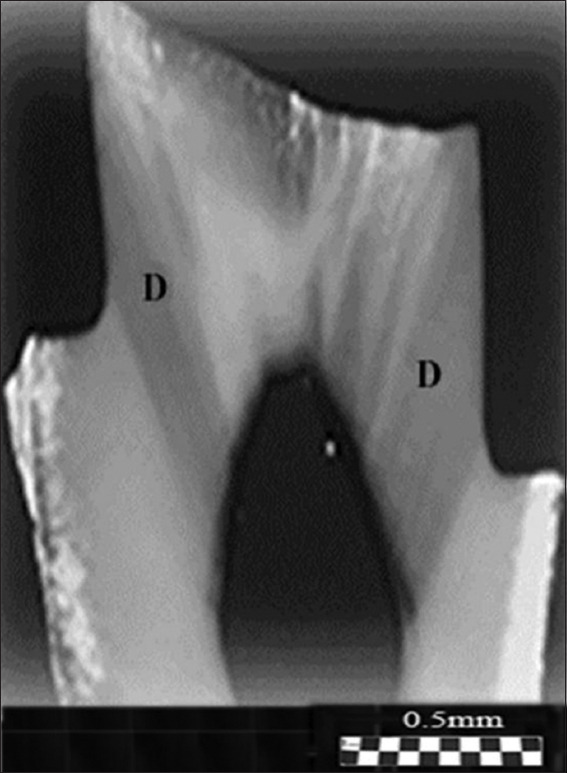
Section of the modal (mesio-occlusal-distal) tooth preparation on the third molar. A dark blue dye was introduced into the pulp chamber under pressure following tooth preparation.^13^ The dark areas of dye penetration (D) indicate that the dentinal tubules of the axial walls are significantly more permeable than those adjacent to the pulp. The dark oval region in the lower part of the image represents the pulp area, which is highly permeable to the dye. Scale bar: 0.5 mm.

Comparison of optical data with the ion diffusion experiment (Figure 11) shows differences in the contrast of observed dentin areas and the specific organization of tubule bundles. The discontinuity of the dark areas of dentin (Figures 13 and 14) is associated with the configuration of the S-beams, consisting of anisotropic tubules that bend from the pulp toward the tips of the tooth tubercles. The dark shading due to dye diffusion (D, left), as shown in Figure 15, indicates that the beam in the left part of the crown is uniform along the axis, whereas the broader beam (D, right) on the right is supplemented by a number of narrow, curved S-beams separated by light gaps.

**Figure 16:**
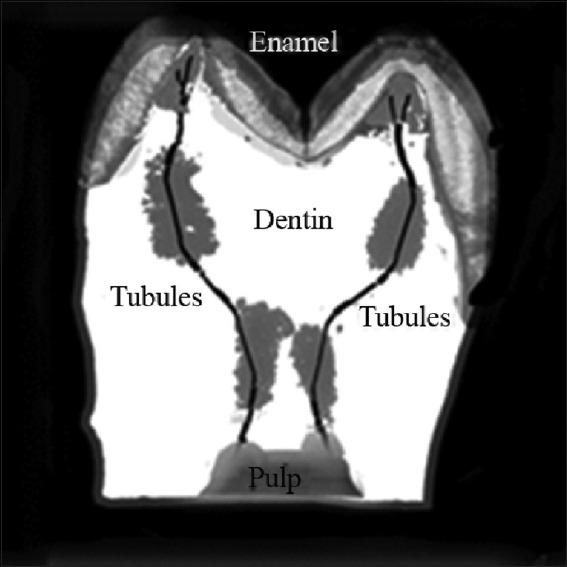
Diagram of a molar section simulating the observed patterns shown in Figure 13. Curved S-beams consisting of anisotropic tubules are schematically depicted to the left and right of the central axis of the tooth. The diagram highlights opaque regions of the section that correlate with areas of S-beam bending of anisotropic tubules, as observed in Figure 13. The dark areas near the S-beams correspond to orientations perpendicular to the E-vector of the incident light.

When the direction of the E-vector is perpendicular to a selected section of an S-beam, the polarized light passing through that section will retain its E-vector direction. As a result, the So beam passing through the S-beam is extinguished by the analyzer, forming dark areas in the photo. In contrast, when the light passes through a bending section of the S-beam, the E vector of the incident light forms an angle with the beam axis. This causes both So and Se beams to arise, introducing a phase difference δ and producing elliptically polarized light. This light is only partially extinguished by the analyzer. The model diagram (Figure 16) illustrates the bending characteristics of two S-beams formed from anisotropic tubules.

Figure 16 schematically shows the inflection areas of both S-beams, where dark regions and light areas, comparable in brightness to the scattered dentin background, are formed. Both S-beams maintain a bending pattern extending from the pulp to the tooth tubercles (Figure 16). The axial symmetry of these S-beams corresponds well with the patterns observed in horizontal sections (Figure 2). Light zones in other areas of the tooth (Figure 16) result from sets of S-beams with a disordered structure.

The alternation of light and dark fragments along the axis of S-bundles allows independent estimation of the linear dimensions of dark areas that represent growth zones in the tooth tubercles, for sections cut perpendicular to the tooth axis. These dimensions correlate with the main pitch and diameters of the curved bundles, which can be similarly estimated in perpendicular sections. In addition, the pitch of a single bundle can be studied to compare parameters across different sections of the same tooth.

Thus, the use of crossed polarizers with low magnification enables complete visualization of entire tooth sections. The studies showed that the dentin structure near the molar tubercles consists of dentin tubules arranged in an axisymmetric pattern. The details of symmetry and asymmetry within these structures reflect changes in the shape of individual tooth tubercles. Analyzing the symmetry or asymmetry of individual growth zones provides additional insight into evolutionary mechanisms in the development of multi-rooted human teeth. The twisting of S-tubes at various distances from the pulp can also be evaluated to compare parameters of adjacent bundles. For different teeth, the symmetrical features of growth zones in the tubercle area (Figures 1 and 2) can be compared with the image in Figure 13. These comparisons show that the patterns of horizontal sections in the crown area vary in detail between teeth.

The organization of tubules observed via polarization optics in vertical sections of individual tooth tubercles appears less structurally pronounced compared to horizontal sections (compare Figures 1, 2, and 8 with Figures 13 and 14). These structural features are influenced by the variability in the number of growth centers of individual tooth tubercles and their shapes (Figures 3 and 11).

The growth centers of individual tooth cusps are influenced by the characteristics of phenotypic development. In future studies, it would be beneficial to analyze vertical and horizontal sections of the same tooth, which would allow for direct comparison of structural data between different planes of sectioning.

## 4. Conclusion

The use of polarized light with slight magnification of the section image allows for simultaneous observation of the entire pattern in full-size sections of the tooth crown. Independent studies of tooth morphology have shown that the complexity of tubercle shape directly reflects the animal’s diet in many mammalian species.^3^,^54^-^56^ These studies have pointed to higher-order, generalizable principles that control the shape and size of teeth. Studies on the structure of regional zones of the tooth have shown that the dentin structure in the vicinity of the central part of the largest tubercles is formed by a set of dentinal tubules arranged in an axisymmetric pattern. As the tubercle shifts from the center, this symmetry is disrupted, a phenomenon most pronounced in smaller tubercles. The details of symmetry–asymmetry fragments reveal specific shape changes in individual tooth tubercles during the phenogenetic type of dentinogenesis. Analysis of the symmetry and asymmetry in individual growth zones of the tooth provides an additional approach to studying the evolutionary mechanisms underlying the formation of multi-rooted human teeth. The combined use of physical methods in this work increases the reliability of the findings and provides additional tools for investigating the evolutionary mechanisms involved in human tooth development.

## Data Availability

The data that support the findings of this study are available from the corresponding author upon reasonable request.
